# Implicit and Explicit Self-Identification as a Drug User in People Who Used Heroin and Methamphetamine

**DOI:** 10.3389/fpsyg.2021.685110

**Published:** 2021-07-02

**Authors:** Jianyong Chen, Meng Zhang, Jifan Zhou, Xinyu Li, Feng Zhang, Mowei Shen

**Affiliations:** ^1^Department of Psychology, Zhejiang Normal University, Jinhua, China; ^2^Department of Psychology and Behavioral Sciences, Zhejiang University, Hangzhou, China; ^3^Department of Psychology, Ningbo University, Ningbo, China

**Keywords:** implicit cognition, self-identity, heroin, methamphetamine, single category implicit association test

## Abstract

Implicit and explicit self-identification as a drug user specific to the substance used (e.g., viewing oneself as a drinker) have been examined, as they relate to that drug use. However, studies have rarely explored whether identifying as a “drug user” differs implicitly and explicitly for people who use different drugs and how this identification relates to drug-use behaviors or abstinence. This study examined implicit and explicit self-identification as a “drug user” and their associations with drug-use behaviors and abstinence in people who used heroin (PWUH) and people who used methamphetamine (PWUM). Forty PWUH and 35 PWUM in a rehabilitation facility completed the single category implicit association test (SC-IAT), which evaluated implicit associations of a “drug user” with “self,” and a measure of explicit self-identification as a “drug user.” Prior drug-use behaviors and current abstinence duration of the participants were assessed. PWUH demonstrated stronger implicit “self + drug user” associations and higher levels of explicit self-identification as a “drug user” than PWUM. A higher frequency of drug use was associated with higher levels of explicit drug-user self-identity, and longer abstinence duration was positively related to stronger implicit “self + drug user” associations in PWUH. The drug type of heroin (vs. methamphetamine) participants used was associated with a higher frequency of use, which, in turn, predicted higher levels of explicit drug-user self-identity. Given that the PWUH group differs from the PWUM group in terms of implicit and explicit self-identification as “drug users,” it would be more appropriate to address drug-user self-identity of individuals according to the substance they use rather than as a collective group.

## Introduction

Self-identification as a drug user can put individuals at risk of initiating, sustaining, and relapsing into drug use (Walters, 1996; Avants et al., 2000; Lindgren et al., 2016b). People who use different types of drugs may differ in their levels of self-identification as a drug user (Avants et al., 1993), justifying the importance of exploring how self-identification as a drug user can affect drug use and treatment that includes identity change based on specific substances. This study investigated self-identification in people who used heroin (PWUH) and people who used methamphetamine (PWUM) to understand drug-use development and treatment in these populations.

In recent years, implicit cognition of drug users has become a major research focus. Implicit cognition involves automatic appraisal of stimuli in terms of their emotional and motivational significance, and it differs from explicit cognition, which includes controlled processes related to conscious deliberations (Wiers and Stacy, 2006; Wiers et al., 2007). According to some theorists, addictive behaviors are a joint outcome of implicit and explicit cognitions (Wiers et al., 2007; Stacy and Wiers, 2010). This opinion is supported by the fact that both implicit and explicit cognitions (e.g., implicit and explicit attitudes toward drugs) reliably correlate with drug use (Rooke et al., 2008). Many studies have used the Implicit Association Test (IAT; Greenwald et al., 1998) to examine implicit cognition of drug users (e.g., implicit self-identification as a drinker; Lindgren et al., 2016b) by measuring the relative strength of associations between target concepts (e.g., a drinker vs. a non-drinker) and attribute categories (e.g., self vs. other). The IAT effects are thought to reflect associations in memory over which individuals have limited control; they may have a unique predictive value because people may not be willing or able to report relevant cognition (De Houwer et al., 2009). Therefore, measures of implicit cognition complement measures of explicit cognition.

Prior studies found that college student drinkers (Gray et al., 2011; Lindgren et al., 2013a,b) and non-student adult drinkers (Frings et al., 2016; Lindgren et al., 2016a; Montes et al., 2018) showed similar implicit associations between “drinker + me” and “non-drinker + me” (Lindgren et al., 2013a,b, 2016a; Frings et al., 2016; Montes et al., 2018), or a greater association of “water + me” as compared with “alcohol + me” (Gray et al., 2011). Use of different labels (i.e., alcohol and water vs. a drinker and a non-drinker) and stimuli (i.e., images of alcohol and water vs. words describing a drinker and a non-drinker) in two target categories of the IAT may explain the different findings of implicit self-identity among the studies (cf. Houben and Wiers, 2006). Nevertheless, implicit self-identification with drinker (or alcohol) consistently and positively predicted unique variance in alcohol consumption and alcohol-related problems after controlling for explicit self-identification with drinker (or alcohol) (Gray et al., 2011; Lindgren et al., 2013a,b, 2016a; Frings et al., 2016) and other implicit alcohol-related associations (Lindgren et al., 2013b). Additionally, compared with college student non-smokers, college student smokers showed stronger implicit associations of “self + smoking,” and their stronger implicit self-identification with smoking was associated with a greater frequency of smoking (Swanson et al., 2001). More recently, a study on young adults who were at risk of drug use has revealed that higher implicit self-identification with drug use predicted more serious concurrent illicit drug use (Horwitz et al., 2019).

There is limited research on implicit and explicit self-identity of individuals who have abstained from drug use for a certain period, especially among PWUH and PWUM. According to the understanding of stigma as a “spoiled identity,” inherited from Goffman (1963), drug use and recovery are viewed as management and even reconstitution of this spoiled identity. Hence, it is fundamental to investigate self-identification as a drug user and its association with prior drug use and current recovery in individuals who have abstained from drugs. One study used the Single Category Implicit Association Test (SC-IAT; Karpinski and Steinman, 2006) to examine implicit associations between self and heroin in people being treated for heroin or alcohol use (Brener et al., 2012). The SC-IAT was adapted from the IAT and could assess implicit associations with a single category (e.g., heroin) without a contrasting category (Brener et al., 2012). PWUH (vs. alcohol users) displayed a stronger implicit association between self and heroin, and higher levels of prior heroin use predicted greater “self + heroin” associations.

However, people who use different drugs may all be given a superordinate label of a “drug user.” Levels of implicit and explicit self-identification as a “drug user” in general may, nevertheless, differ in users of different substances, which may be ascribed to their different drug-use patterns. Long periods of regular use characterize heroin addiction, while methamphetamine is generally used episodically at lower levels (e.g., weekend users) (Hser et al., 2008b). Regular drug users are more likely to have a drug-user social network (Simons-Morton and Chen, 2006; Creemers et al., 2010), which possibly strengthens their self-identification. An earlier study has shown that heroin-using police detainees were more likely to self-identify as drug dependent than cocaine-, amphetamine-, and cannabis-using police detainees, and the probability of self-identifying as drug dependent was associated with a drug type, as well as frequency and longevity of use (Langfield and Payne, 2020). Taken together, PWUH may differ from PWUM in terms of the strength with which they self-identify as a “drug user,” and such differences may be associated with different drug-use patterns (e.g., frequency of use) between the two groups.

This study aimed to compare the strength of implicit and explicit self-identification as a “drug user” between PWUH and PWUM and investigate the effects of drug-use patterns on the strengths of implicit and explicit drug-user self-identity. It was hypothesized that (1) PWUH would indicate stronger implicit and explicit self-identification as a “drug user” than PWUM, (2) higher levels of prior drug use would predict higher strengths of implicit and explicit self-identification among PWUH and PWUM groups, and (3) stronger implicit and explicit self-identification as a “drug user” among PWUH compared with PWUM may be associated with higher levels of drug-use behaviors (e.g., frequency of use). We also explored the associations between abstinence duration in a rehabilitation facility and implicit and explicit self-identity instead of offering a hypothesis because there was less certainty about how these variables would be related.

## Materials and Methods

### Participants

Forty participants with a history of heroin use and 35 participants with a history of methamphetamine use at a rehabilitation facility in Zhejiang province, China participated in this study (see Table 1). Participation was voluntary, and no incentives were provided. The participants in each group used either heroin or methamphetamine only (i.e., PWUH had no history of methamphetamine use, and PWUM had not used heroin). Only male participants were recruited because most illicit drug users in this facility were male. The participants met the DSM-IV criteria for heroin or methamphetamine dependence and had no diagnosed history of mental disease or brain injury before participation. They had not received drug substitution treatment or other medications that influence the central nervous system. Information on their drug-use behaviors (i.e., months of use, times of use per month, quantity of use per month, and number of relapses) prior to entering the rehabilitation facility was collected (see Table 1; Chen et al., 2015; Zhang et al., 2015). The participants abstained from drug use after entering the facility. The abstinence duration was based on the length of their stay at the facility, and their abstinence duration ranged from 3.60 to 25.13 months (*M* = 12.27, *SD* = 6.36). To ensure that the patients at the facility do not have access to drugs, they were not allowed to leave the rehabilitation facility and were subjected to routine urine tests. Although the participants with a drinking and/or smoking history were included, none of them had a history of heavy drinking or smoking. All the participants were native Chinese speakers and right-handed. Analyses of the primary demographic data and clinical characteristics are reported in Table 1.

**Table 1:** Demographic characteristics of and differences between people who used heroin (PWUH) and people who used methamphetamine (PWUM).

**Characteristic**		**PWUH (*n* = 40)**	**PWUM (*n* = 35)**	**Significance**
Age (*M* ±*SD*)		32.60 ± 7.33	32.86 ± 7.96	*t*(73) = −0.15, *p* = 0.89
Education level^a^ (number/percentage)	Primary school	13/32.5%	3/8.6%	χ^2^(3) = 7.43, *p* = 0.059
	Junior high school	23/57.5%	25/71.4%	
	Senior high school	4/10%	6/17.1%	
	College	0	1/2.9%	
Abstinence duration (*M* ±*SD*, month)		12.89 ± 6.58	11.58 ± 6.11	*t*(73) = 0.89, *p* = 0.38
Route of administration (number/percentage)	Inhalation	30/75%	35/100%	χ^2^(2) = 10.10, *p* = 0.006
	Injection	7/17.5%	0	
	Both	3/7.5%	0	
Months of drug use (*M* ±*SD*)		101.33 ± 72.49	76.09 ± 46.04	*t*(66.93) = 1.82, *p* = 0.073
Times of use per month (*M* ±*SD*)		69.50 ± 45.12	21.83 ± 15.77	*t*(49.54) = 6.26, *p* < 0.001
Quantity of use per month (*M* ±*SD*, gram)		21.03 ± 19.59	9.61 ± 8.23	-
Lifetime incidences of relapse^b^ (*M* ±*SD*)		4.23 ± 4.85	5.14 ± 6.93	*t*(73) = −0.67, *p* = 0.51
Past other drug dependence (number)		0	0	-

a *Participants who were coded as primary school referred to those who had completed the 6 years of elementary education, those coded as junior high school referred to those who had completed the first 3 years of secondary education, those coded as senior high school indicated that they had completed the full 6 years of secondary education, and those coded as college referred to those that had completed the full 4 years of higher education*.

b *A relapse was defined as ≥1 day of heroin or methamphetamine use preceded by an abstinence period of 30 days or longer during which the participants did not use heroin or methamphetamine (c.f. Mckay et al., 1996)*.

### Measures

#### Single Category Implicit Association Test

Five drug-user-related words (Chinese language equivalents of “drug user,” “druggie,” “addict,” “junkie,” and “substance user”) were used as target words in the SC-IAT. Drug-user-related words were gathered based on a combination of earlier studies (Zogmaister et al., 2013; von Hippel et al., 2018) and a semi-structured interview with 27 male PWUH and 25 PWUM from the same rehabilitation facility who did not participate in the formal study. They were asked to rate each of the words, using a Likert scale, ranging from 1 (extremely irrelevant) to 5 (extremely relevant) on the extent to which the word is representative of a drug user. The target words were determined by their scores, approaching the high ends on the scale (the PWUH group: 3.48–4.30 [*M* = 4.01, *SD* = 0.31] and the PWUM group: 3.56–4.28 [*M* = 4.06, *SD* = 0.50]). Eight self-related words (Chinese language equivalents of “myself,” “self,” “mine,” “me,” “my,” “personal,” “we,” and “us”) and eight other-related words (Chinese equivalents of “other,” “others,” “his,” “they,” “them,” “their,” “theirs,” and “those”) were used as attribute words (Cai, 2003; Hu, 2009).

#### Explicit Measure of Drug-User Self-Identity

The drug-user-related words used in the SC-IAT were employed to construct an explicit measure of drug-user self-identity (cf. Wiers et al., 2002). The same words were used in the SC-IAT and explicit measure of self-identity to create high structural similarity and to ensure the comparability of the implicit and explicit measures (Serenko and Turel, 2019). The participants were instructed to rate their agreement with statements (i.e., “I am a drug user,” “I am a druggie,” “I am an addict,” “I am a junkie,” and “I am a substance user”) using a 7-point Likert scale (1 = *extremely disagree*, 7 = *extremely agree*). Hence, the explicit measure of drug-user self-identity was composed of five items, and higher scores indicated more self-identification as a drug user. The internal reliability of the explicit measure in this study was reasonably good (the PWUH group = 0.84; the PWUM group = 0.72).

### Procedure

First, the SC-IAT was presented, followed by the explicit measure of drug-user self-identity. The two measures were administered *via* Inquisit 4.0 (2015). The SC-IAT consisted of two stages, and each stage included two blocks. For half of the participants in each group, the first stage began with a block of 24 practice trials that required the participants to press “E” on a computer keyboard in response to *self-* or *drug-user-related* words or “I” in response to *other related* words. The second block required participants to complete 72 test trials in which the assignment was identical to that of the first block. Next, the second stage began with a block of 24 practice trials in which they pressed the “E” key to respond to the *self-related* words and the “I” key to respond to the *other-* or *drug-user-related* words. Finally, the participants completed the fourth block of 72 test trials in which the assignment was identical to that of the third block. The order of the two stages was reversed for the other half of the participants. Instructions for the following blocks were presented before each block. The categorical labels (i.e., self, drug user, and other) were appropriately displayed in the top-left or top-right corner of the screen during the practice and test blocks. The participants were instructed to classify the words according to the categorical labels and respond as quickly and accurately as possible. Implicit self-identification as a drug user was inferred by the relative ease (i.e., speed) with which “self” vs. “other” attributes were paired with the target concept.

Each target or attribute word appeared at the center of the computer screen and remained there until the participants responded. Following each response, the feedback was displayed. A green *O* appeared beneath the word for 150 ms if the participants responded correctly. A red *X* was displayed under the word if the participants gave an incorrect response. The *X* and words were displayed until the participants corrected their responses. The following trial began 250 ms after each correct response.

After the SC-IAT, the participants were instructed to complete the explicit measure in which they indicated the extent to which they agreed with each statement. Each statement was displayed at the center of the screen in a fixed order. The participants rated each statement by clicking one of the seven number buttons under the statement. A label was presented on each button (e.g., “extremely disagree” on the number “1” button). After a choice was made, the result was recorded automatically, and the next statement appeared on the screen.

### Data Reduction and Analysis

In accordance with Karpinski and Steinman's (2006) study, the *D*-score algorithm was used for the SC-IAT data. Reaction times slower than 10,000 ms and faster than 350 ms were eliminated. The participants whose error rates for test blocks (i.e., blocks 2 and 4) exceeded 20% or who exhibited mean latency over 2,000 ms were excluded from the analyses (Greenwald and Farnham, 2000), resulting in the elimination of four PWUH and one PWUM. An error penalty for erroneous responses was not included in the SC-IAT because the participants could not proceed to the next stimulus until they made a correct response (Greenwald et al., 2003; Lane et al., 2007). The reaction times for each test block were then averaged, and a difference score was calculated by subtracting the mean of the “self + drug user” block from the mean of the “other + drug user” block. Next, the difference score was divided by the SD of all included reaction times from blocks 2 and 4 (i.e., *D*-score). Positive *D*-scores indicated stronger associations between “self” and “drug user” relative to “other” and “drug user.” Negative *D*-scores indicated greater associations between “other” and “drug user” relative to “self” and “drug user.” A*D*-score of zero indicated a neutral association.

To test the difference in SC-IAT *D*-scores and ratings of explicit self-identity measure between the PWUH and PWUM groups, one-way univariate analyses of covariance were conducted with group (heroin, methamphetamine) as a between-subjects variable and drug-use behaviors, abstinence duration, age, and education level as covariates. No significant effects of the covariates were found (*p*s > 0.11). One-sample *t*-tests were used to analyze whether the scores of PWUH or PWUM on the explicit measure or SC-IAT *D*-scores differed from the midpoint of the explicit measure (i.e., 4) or the point at which the participants had neutral associations (i.e., 0) (Karpinski and Steinman, 2006; Bardin et al., 2014). Pearson bivariate correlations between SC-IAT *D*-scores, ratings of explicit drug-user self-identity, drug-use behaviors, and abstinence duration were calculated separately for each group. To avoid the risk of a type I error due to calculating numerous correlation coefficients, multiple regression analyses were also performed (Curtin and Schulz, 1998). Specifically, SC-IAT *D*-scores and ratings of explicit self-identity were regressed onto sociodemographic features (i.e., age and education level), drug-use behaviors, and abstinence duration. To explore whether a drug type indirectly influenced implicit and explicit drug-user self-identity *via* drug-use behaviors or abstinence duration, we tested mediator models *via* SPSS and the macro PROCESS (Hayes, 2018). The drug type (the PWUM group was coded 0, and the PWUH group was coded 1) was entered as an independent variable (X), drug-use behaviors (i.e., months of use, times of use per month, and number of relapses) and abstinence duration were entered as mediators (M), and ratings of explicit drug-user self-identity or SC-IAT *D*-scores were the dependent variables (Y). The quantity of use per month was not included in the model because heroin and methamphetamine do not produce the “high” effect for the same duration. We controlled for age and education level. The estimates of 95% confidence intervals of standardized effects were calculated using 1,000 bootstrapped samples.

## Results

### Implicit and Explicit Drug-User Self-Identity

Results of SC-IAT *D*-scores revealed that PWUH (*M* = 0.04, *SD* = 0.31) showed stronger implicit self-identification with “drug users” than PWUM (*M* = −0.16, *SD* = 0.28), [*F*_(1, 61)_ = 4.71, *p* = 0.034, *η*^2^_*p*_ = 0.07]. Moreover, the PWUH group showed no significant difference between “drug user + self” and “drug user + other” associations, *t*(35) = 0.71, *p* = 0.48, Cohen's *d* = 0.12, while the PWUM group exhibited evidence of associating “drug user” with “other” more strongly than with “self,” *t*(33) = 3.33, *p* = 0.002, Cohen's *d* = 0.58.

Results of ratings on the explicit measure showed that the PWUH group (*M* = 5.32, *SD* = 1.20) indicated higher levels of self-identification as a drug user compared with the PWUM group (*M* = 4.13, *SD* = 1.07), [*F*_(1,66)_ = 5.41, *p* = 0.023, *η*^2^_*p*_ = 0.08]. The ratings of the PWUH on the explicit measure were significantly higher than the midpoint of this measure, *t*(39) = 6.93, *p* < 0.001, Cohen's *d* = 1.11, while the ratings of PWUM were not significantly different from the midpoint, *t*(35) = 0.69, *p* = 0.49, Cohen's *d* = 0.12.

### Associations Between Drug-Use Behaviors, Abstinence Duration, and Implicit and Explicit Drug-User Self-Identity Among the PWUH Group

Bivariate correlations linking drug-use behaviors, abstinence duration, and implicit and explicit drug-user self-identity are displayed in Table 2. For the PWUH group, times of heroin use per month and months of heroin use were both significantly positively correlated with ratings of explicit drug-user self-identity (*r*s > 0.32, *p*s < 0.047). The quantity of heroin use per month was positively correlated with explicit self-identity ratings, although this correlation was not statistically significant (*r* = 0.29, *p* = 0.068). Moreover, abstinence duration significantly positively correlated with SC-IAT performance (*r* =0.40, *p* =0.02). No other significant correlations were found (0.11 < *r* < 0.25, *p*s > 0.12). In the multiple regression model for explicit drug-user self-identity, times of use per month positively predicted explicit self-identity ratings among PWUH (*β* = 0.36, *p* = 0.032), while other types of drug-use behaviors and abstinence duration did not (*β*s < 0.15, *p*s > 0.10). Meanwhile, abstinence duration significantly positively predicted SC-IAT *D*-scores among PWUH (*β* = 0.42, *p* = 0.024), whereas drug-use behaviors did not (*β*s < 0.25, *p*s > 0.10). Additionally, performance on the SC-IAT was not significantly correlated with ratings of explicit drug-user self-identity measure (*r* = 0.28, *p*s > 0.10).

**Table 2:** Pearson's correlations for implicit and explicit self-identity as a drug user and drug-use behaviors and abstinence duration.

	**1**	**2**	**3**	**4**	**5**	**6**	**7**
1. Implicit drug-user identity	-	**0.28** (−0.12)	**0.11** (−0.19)	**0.17** (−0.04)	**0.13** (−0.29)	**0.20** (−0.03)	0.40^*^ (−0.12)
2. Explicit drug-user identity		-	0.32^*^ (0.26)	0.40^*^ (0.15)	**0.29**^**†**^ (0.06)	**0.22** (0.03)	**0.24** (0.02)
3. Months of heroin or methamphetamine use			-	**0.27** (0.11)	0.35^*^ (0.22)	0.57^***^ (0.50^**^)	**−0.14** (−0.23)
4. Times of use per month				-	**0.30**^†^ (0.32^†^)	**0.16** (−0.12)	**0.25** (−0.14)
5. Quantity of use per month					-	**0.20** (−0.001)	**0.18** (−0.08)
6. Lifetime incidences of relapse						-	**−0.18** (−0.35^*^)
7. Abstinence duration							-

*The numbers in bold were calculated from data of people who used heroin, while those in parenthesis were calculated from data of people who used methamphetamine*.

† *p < 0.07*,

* *p < 0.05*,

** *p < 0.01*,

*** *p < 0.001*.

### Associations Between Drug-Use Behaviors, Abstinence Duration, and Implicit and Explicit Self-Identity Among the PWUM Group

For the PWUM group, there were no significant correlations between drug-use behaviors, abstinence duration, and implicit and explicit drug-user self-identity (−0.29 < *r* < 0.27, *p*s > 0.10). In addition, performance on the SC-IAT was not significantly correlated with ratings of explicit drug-user self-identity measure (*r* = −0.12, *p*s > 0.50).

### Analyses of the Mediating Roles of Drug-Use Behaviors and Abstinence Duration

**Figure 1:**
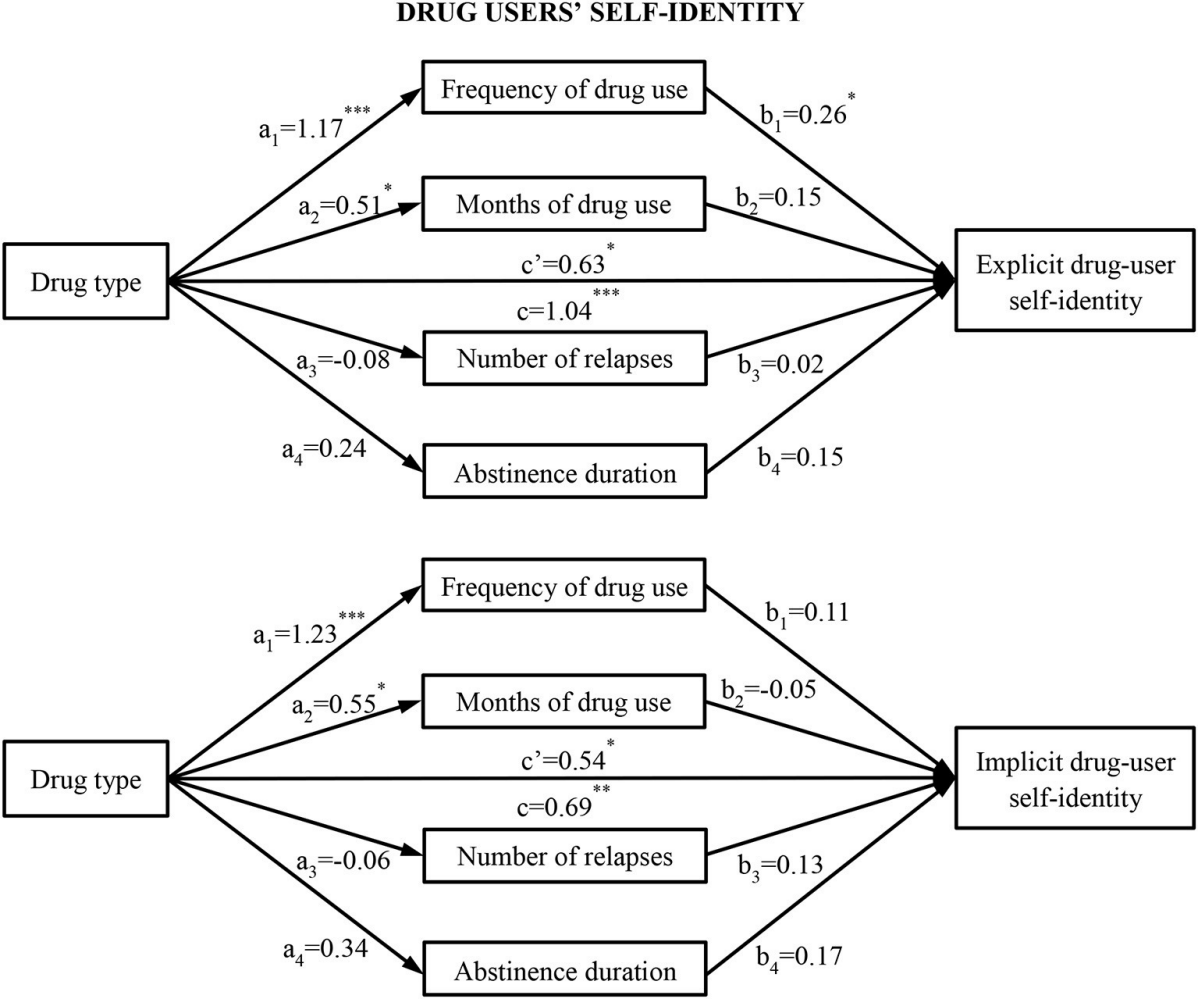
Drug-use behaviors and abstinence duration as mediators between drug type (i.e., heroin and methamphetamine) and implicit and explicit drug-user self-identity. The (c') value represents the direct effect of the drug type on implicit and explicit drug-user self-identity after the mediators were included. The (c) value represents the effect of the drug type on implicit and explicit self-identity before the inclusion of the mediators. ^*^p < 0.05, ^**^p < 0.01, ^***^p < 0.001.

In the mediation model for explicit drug-user self-identity, drug type demonstrated a significant positive association with explicit self-identity ratings (*b* = 1.04, *p* < 0.001, 95% CI [0.62, 1.46]), with PWUH (vs. PWUM) having higher explicit self-identity ratings. The drug type significantly positively predicted times of drug use per month (*b* = 1.17, *p* < 0.001, 95% CI [0.77, 1.57), and months of drug use (*b* = 0.51, *p* = 0.02, 95% CI [0.08, 0.93]), while drug type did not predict number of relapses and abstinence duration (*b*s < 0.24, *p*s > 0.30, 95% CI [−0.24, 0.72] and [−0.56, 0.41], respectively). The times of use per month positively predicted explicit self-identity ratings (*b* = 0.26, *p* = 0.04, 95% CI [0.01, 0.51]), while months of use, number of relapses, and abstinence duration did not (*b*s < 0.15, *p*s > 0.16, 95% CI [−0.12, 0.41], [−0.21, 0.25], and [−0.06, 0.35], respectively). The direct effect of drug type on explicit self-identity ratings was significant when drug-use behaviors and abstinence duration were included in the model (*b* = 0.63, *p* = 0.01, 95% CI [0.14, 1.12]). The indirect effect of drug type on explicit self-identity ratings through times of use per month (*ab*_cs_ = 0.30, 95% CI [0.01, 0.61]) was significant, while the indirect effects through other types of drug-use behaviors and abstinence duration were not significant (all *ab*_cs_ < 0.08). Hence, drug type may directly positively predict explicit self-identity ratings while exerting an indirect influence on explicit self-identity ratings through the frequency of use per month as well (Figure 1).

In the mediation model for implicit self-identity, drug type demonstrated a significant positive association with SC-IAT *D*-scores (*b* = 0.69, *p* = 0.005, 95% CI [0.21, 1.17]), indicating that PWUH (vs. PWUM) had stronger implicit associations between “self” and “drug user.” The drug type significantly positively predicted times of use per month (*b* = 1.23, *p* < 0.001, 95% CI [0.81, 1.65]) and months of use (*b* = 0.55, *p* = 0.01, 95% CI [0.12, 0.97]), while drug type did not predict abstinence duration and number of relapses (*b*s < 0.34, *p*s > 0.17, 95% CI [−0.16, 0.83] and [−0.57, 0.45], respectively). Abstinence duration and drug-use behaviors did not significantly predict SC-IAT *D*-scores (*b*s < 0.18, *p*s > 0.17, 95% CI [−0.08, 0.42], [−0.19, 0.40], [−0.38, 0.27], and [−0.14, 0.40], respectively). The direct effect of drug type on SC-IAT *D*-scores was significant when abstinence duration and drug-use behaviors were included in the model (*b* = 0.54, *p* = 0.03, 95% CI [0.05, 1.04]). The indirect effects of drug type on SC-IAT *D*-scores through abstinence duration and drug-use behaviors were not significant (all *ab*_cs_ < 0.13). Therefore, drug-use behaviors and abstinence duration did not mediate the relation between drug type and SC-IAT *D*-scores.

## Discussion

People who used heroin had higher levels of implicit and explicit self-identification as a drug user compared with PWUM. This finding resembles the results from previous studies (Brener et al., 2012; Langfield and Payne, 2020). For example, in Australia, heroin-using police detainees were more likely to self-identify as drug dependent than cocaine-, amphetamine-, and cannabis-using police detainees (Langfield and Payne, 2020), and PWUH had stronger implicit associations between self and heroin than alcohol users (Brener et al., 2012). Thus, the present study is consistent with prior research on self-identification with a drug user among PWUH compared with people who use other substances, despite having abstained from drug use for a period of time.

This difference in self-identification with a “drug user” between the PWUH and PWUM groups may be explained by their different drug-use patterns. Researchers have proposed that self-identification with drug dependence was most common among higher frequency users and for those who had used drugs over a longer period of time (Langfield and Payne, 2020). Consistent with this proposition and other studies with American samples (Hser et al., 2008a,b), we found that the PWUH group used the drug more frequently and had a longer history of use than the PWUM group. Because regular use over a long period of time can lead to higher likelihood of exposure to social networks of drug-using peers (Simons-Morton and Chen, 2006; Creemers et al., 2010), and higher levels of drug use can strengthen the self-identification as a drug user among PWUH. Additionally, the typical image of a drug user in the media and films in China has generally been PWUH rather than PWUM (e.g., users sniffing up white powder or injecting the fluid made from white powder to attenuate their withdrawal symptoms) (Li, 2020). Combined with the findings that heroin is considered more dangerous and harmful than methamphetamine (Zou et al., 2012), PWUH may experience more rejection and unfair treatment from the general public than PWUM, possibly increasing their identification as “drug users” in-group to fulfill their need for belongingness and acceptance (Branscombe et al., 1999).

This study further investigated the relationships between drug-use behaviors, abstinence duration, and implicit and explicit drug-user self-identity, separately for PWUH and PWUM groups. For the PWUH group, higher levels of drug use (especially for times of use per month) were associated with stronger explicit self-identification as a drug user. This finding is in line with studies in which explicit self-identity was positively associated with drug-use behaviors in alcohol (Lindgren et al., 2013b, 2016c), nicotine (Dupont et al., 2015), and cannabis (Pearson et al., 2017; Blevins et al., 2018) users. However, it is unclear whether self-identity by users predicts future use. Prior studies have shown that explicit self-identity by college students predicted their future alcohol consumption (Lindgren et al., 2016c), but self-identity by young adult drug users was not predictive of future use (Horwitz et al., 2019). A longitudinal study is necessary to examine whether self-identification as a drug user affects future use in PWUH. For the PWUM group, drug-use behaviors were not significantly correlated with explicit drug-user self-identity. This result is inconsistent with our hypothesis; it is unclear what factors may account for it.

The mediation model for explicit drug-user self-identity provides a better understanding of the self-identity of drug users by establishing that the differences in explicit self-identification with drug users between PWUH and PWUM might be ascribed to drug-use behavior (i.e., frequency of use). Previous studies have shown that PWUH differed from PWUM in drug-use behaviors. For example, Hser et al. (2008b) revealed that PWUH showed a higher frequency of drug use per month than PWUM over a 10-year period. In addition, Wang et al. (2017) showed that PWUH had a different drug addiction process compared with PWUM. Specifically, PWUH had shorter transitions from the onset of drug use to the first drug craving (19.5 vs. 50.0 days), regular use (30.0 vs. 60.0 days), and compulsive use (50.0 vs. 85.0 days) than PWUM. More frequent drug use, faster transitions to drug craving, regular use, and compulsive use among PWUH compared with those of PWUM may increase the differences in self-identification as a drug user between the two populations. Considering that PWUH differ from PWUM in drug-use patterns, drug addiction process, and strength of implicit and explicit drug-user self-identity, it is necessary to address their self-identity through educational and cognitive retraining interventions based on the substance used but not as a collective group.

It was noteworthy that the associations between drug-use behaviors and implicit associations of “self + drug user” were not found in this study. These results are inconsistent with prior studies on alcohol (Gray et al., 2011; Lindgren et al., 2013b), nicotine (Swanson et al., 2001), and heroin (Brener et al., 2012) users. The inconsistency may result from the use of different implicit measures (e.g., IAT vs. SC-IAT) to investigate different aspects of implicit self-identity-related associations (e.g., “self + alcohol” associations vs. “self + drug user” associations). However, abstinence duration positively correlated with implicit associations of “self + drug user” in the PWUH group. Although this opposes prior findings (McIntosh and McKeganey, 2000; Dingle et al., 2015), we speculate that the strengthening of associations between “self” and a “drug user” might take place during rehabilitation treatment for the PWUH group. The PWUH group in this study was under a mandatory detoxification program in the rehabilitation context. Such a context might place individuals in a social network of users and situate their drug-user identity in the spotlight (Cain, 1991; Reith and Dobbie, 2012), and this might prevent them from building social networks with people supporting their goal to quit using drugs and hinder the repair of their spoiled identity. As such, this finding might complement the extant studies and be helpful to understand drug-use development and treatment in the PWUH group from an implicit self-identity perspective. However, it is also possible that such a correlation could be found in the PWUH group before entering the rehabilitation facility. Additionally, interpersonal support or social networks of the participants outside the rehabilitation facility could potentially affect the formation of self-identification as a “drug user” and its relationship with abstinence duration. It is relatively difficult to obtain such information because the participants were not allowed to leave the facility in this study. Future studies are needed, using a longitudinal design that includes relevant factors such as social networks and interpersonal support of users, to test these possibilities.

The mediation analyses for implicit drug-user self-identity revealed that the drug type that heroin (vs. methamphetamine) participants used predicted higher levels of implicit drug-user self-identity, and drug-use behaviors and abstinence duration did not mediate the relationship between drug type and implicit self-identity. These findings suggest that drug-use behaviors and abstinence duration do not seem to contribute to PWUH-PWUM differences in strengths of implicit self-identification with a “drug user.” Our study demonstrated that drug type only had a significant indirect effect on explicit, but not implicit, drug-user self-identity *via* drug-use behavior (i.e., frequency of use). Some dual-process models of social cognition (Wilson et al., 2000; Strack and Deutsch, 2004) and studies (Fazio et al., 1995; Dovidio et al., 1997) indicated that automatic attitudes relate more to spontaneous than to deliberately controlled responses. The information on drug-use behaviors was collected through self-reporting of the participants in this study. The self-reported drug-use behaviors might be substantially associated with explicit rather than implicit measures of drug-user self-identity and mediated the relation between drug type and explicit self-identity.

It is important to note that, in this study, the PWUM group had greater implicit associations of “drug user + other” when compared with “drug user + self.” A previous study found that many PWUM (42.8%) thought that methamphetamine was not addictive, and a large majority (80.4%) believed that they could control their use of methamphetamine (Zou et al., 2012). Moreover, the PWUM group who were involved in the rehabilitation program likely compared themselves with other people (e.g., the PWUH group) in the same program and, consequently, were less likely to self-identify implicitly as drug users. Additionally, we found that the implicit drug-user self-identity had negative correlations with explicit drug-user self-identity, drug-use behaviors, and abstinence duration in the PWUM group. Although these correlations were not significant, we speculate that the negative correlations might be ascribed to the possibility that the PWUM group tends to not self-identify implicitly as “drug users” and to dissociation between implicit and explicit measures (Wiers et al., 2002). It is possible that the PWUM group does not self-identify implicitly as “drug users,” although they have experienced a long history of drug use, received rehabilitation treatment, and had neutral explicit self-identification with “drug users.” Future studies are needed to replicate the present findings. Finally, studies on alcohol (Wiers et al., 2002) and nicotine (Swanson et al., 2001) users have shown that correlations between implicit and explicit measures are generally weak. Combined with our results, implicit and explicit measures may help understand the different aspects of drug-user self-identity.

This study has some limitations. First, its cross-sectional design precludes causal inferences between drug-use behaviors, abstinence duration, and implicit and explicit drug-user self-identity. Further research with both groups is warranted to clarify the directionality of the relationships. Second, this study only included the participants during the period of rehabilitation treatment. Future studies should explore implicit and explicit self-identification in drug users who are in different contexts or in various places along the drug use/dependence continuum. Third, our small sample size likely restricted the possibility of identifying significant associations between implicit and explicit self-identity and drug-use patterns. Additionally, given that heroin and methamphetamine use is characterized by different drug-use patterns (e.g., high vs. low frequency of use) (Hser et al., 2008b), it is difficult to thoroughly disentangle the effects of drug type and drug-use behaviors on the differences of drug-user self-identity between the PWUH and PWUM groups. It is imperative to interpret our results with caution, and future studies are needed to replicate our findings. Finally, this study only included male participants, and the results may not be generalizable to female patients in substance-use treatment.

## Conclusion

In summary, the PWUH group exhibited stronger implicit associations between “self” and a “drug user” and more endorsement of explicit drug-user self-identity than the PWUM group. Higher frequency of use was associated with higher levels of explicit self-identification as a “drug user,” and longer abstinence duration was associated with stronger implicit associations between “self” and a “drug user” in the PWUH group but not in the PWUM group. Additionally, the type of drug individuals used might have exerted an indirect influence on the formation of their explicit drug-user self-identity through drug-use behaviors. These findings may be relevant for relapse prevention and interventions with men who use heroin or methamphetamine.

## Data Availability Statement

The raw data supporting the conclusions of this article will be made available by the authors, without undue reservation.

## Ethics Statement

The studies involving human participants were reviewed and approved by the ethics board of the Department of Psychology and Behavioral Sciences at Zhejiang University. The patients/participants provided their written informed consent to participate in this study.

## Author Contributions

All authors listed have made a substantial, direct and intellectual contribution to the work, and approved it for publication.

## Conflict of Interest

The authors declare that the research was conducted in the absence of any commercial or financial relationships that could be construed as a potential conflict of interest.
